# Restoring Severe Adult Tooth Wear Through Interdisciplinary Approach: A Case Report

**DOI:** 10.1155/crid/7288542

**Published:** 2026-05-26

**Authors:** P. J. Un, D. C.-V. Ong, Jamie Foong

**Affiliations:** ^1^ Private Practice, Hervey Bay, Queensland, Australia; ^2^ School of Dentistry, University of Queensland, Brisbane, Queensland, Australia, uq.edu.au; ^3^ Discipline of Orthodontics, School of Dentistry, University of Queensland, Brisbane, Queensland, Australia, uq.edu.au; ^4^ Private Practice, Townsville, Queensland, Australia; ^5^ Private Practice, Specialist Prosthodontics, Brisbane, Queensland, Australia

**Keywords:** interdisciplinary management, occlusal vertical dimension, orthodontic treatment, severe tooth wear

## Abstract

Comprehensive diagnosis and interdisciplinary collaboration are often required to effectively manage patients presenting with severe tooth wear. A combination of restorative and orthodontic treatment can provide pleasing aesthetic and functional outcomes through increasing the occlusal vertical dimension and appropriate tooth alignment.

## 1. Introduction

Generalised tooth wear in adult patients can be caused by many factors, which include parafunctional habits, acidic corrosion/erosion and/or malocclusion. Bruxism is a voluntary or involuntary parafunctional activity, which may occur during the day and/or night. Bruxism is defined as grinding and/or clenching of teeth together during non‐functional movement of the jaw [1]. This parafunctional activity may be associated with significant damage to the dental, periodontal and musculoskeletal structures. Gastroesophageal reflux disease (GERD) and a highly acidic diet are common contributing factors in tooth erosion.

Tooth wear often results in a reduction in the vertical height of the crown, which can lead to numerous negative sequelae. Patients with severe tooth wear often become more self‐conscious of the aesthetic appearance of their teeth, and progressive tooth wear may also result in compromised dental structure, pain and/or sensitivity, altered speech and mastication difficulties. These problems will commonly motivate patients to seek treatment [2]. Because of multifactorial aetiology associated with severe tooth wear, comprehensive diagnosis and interdisciplinary collaboration is likely to be required to provide effective and long‐term management. This case report aims to showcase how a comprehensive diagnosis in conjunction with appropriate interdisciplinary management can provide a pleasing outcome despite the considerable pre‐existing tooth wear and loss of occlusal vertical dimension (OVD).

## 2. Case Report

A 33‐year‐old medically healthy patient was referred for prosthodontic and orthodontic consultations. The presenting complaint was the progressive loss of tooth structure over the past few years. Upon clinical examination, there was no abnormality detected in the temporomandibular joints. The muscles of mastication were well developed and there were noticeable bilateral crenulations on the buccal mucosa adjacent to the occlusal plane. The patient demonstrated group function guidance during right and left lateral excursive movements of the mandible primarily due to worn canines and overall tooth wear. Isolated contact on tooth 48 was noted during protrusion of the mandible. Generalised gingival recession was detected, along with gingival inflammation associated with teeth 12 and 21. There was evidence of erosion on multiple teeth (i.e., from 14 to the 24, 34 to the 36 and 44 to the 46), with the maxillary incisors demonstrating the most significant wear. The maxillary incisors were reduced in vertical crown height by approximately 50%. Despite this wear, the maxillary anterior teeth remained vital and responded positively to CO_2_ cold testing. The significant wear of the maxillary anterior teeth affected the patient’s speech and caused temperature sensitivity during food and drink intake. Over‐eruption of the teeth had also occurred, resulting in a lack of prosthetic space for prospective restorations. Percussion and CO_2_ cold test were done on tooth 17, 25, 36 and 37 to check on pulp vitality and any signs of periapical pathosis. Based on the negative response to percussion and positive to cold test, the teeth had vital pulp with no significant periapical pathology.

The orthodontic diagnostic assessment revealed a Class I molar and canine relationship and mild mandibular intra‐arch crowding. An edge‐to‐edge anterior occlusion was noted, presumably associated with the significant pre‐existing tooth wear and attrition (Figure 1). The panoramic radiograph showed that all 32 permanent teeth, excluding the 47, were present and there were large restorations in teeth 25, 36 and 37 (Figure 2a). The root morphology and the relative lack of enamel loss present on the terminal tooth in the fourth quadrant led to the assumption that the 47 was removed previously and the 48 had erupted favourably into the mandibular arch. Caries was noted in tooth 17 occlusal surface and the mesial aspect of tooth 11. A lateral cephalogram (Figure 2b) and subsequent analysis demonstrated a mild Class III skeletal relationship base with brachyfacial vertical proportions and slight retroclination of the maxillary incisors. This retroclination was likely the result of the progressive and extensive loss of maxillary anterior tooth structure, both vertically and interproximally. A history of long‐term acidic beverage intake, smoking and tooth grinding were reported. Because of occupational requirements, the patient spent a significant amount of time outdoors, in a relatively dusty environment. The relevant orthodontic diagnostic findings are summarised in Table 1, along with a prioritised problem list and ideal treatment objectives (Table 2). The sequence of the proposed interdisciplinary treatment was discussed in detail between the clinicians involved and with the patient (Table 3).

**Figure 1:**
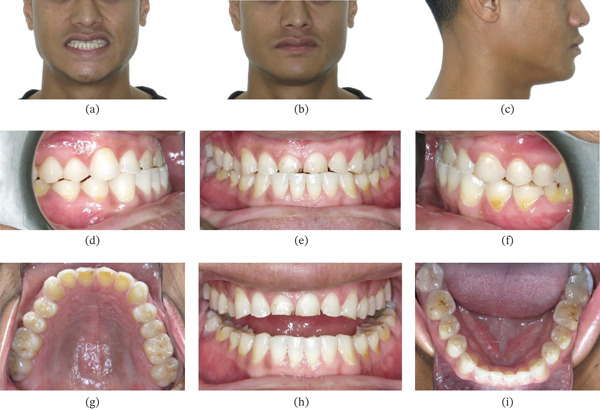
(a–i) Pre‐treatment photographs of a 33‐year‐old patient with significant generalised tooth wear and an edge‐to‐edge anterior occlusion.

**Figure 2:**
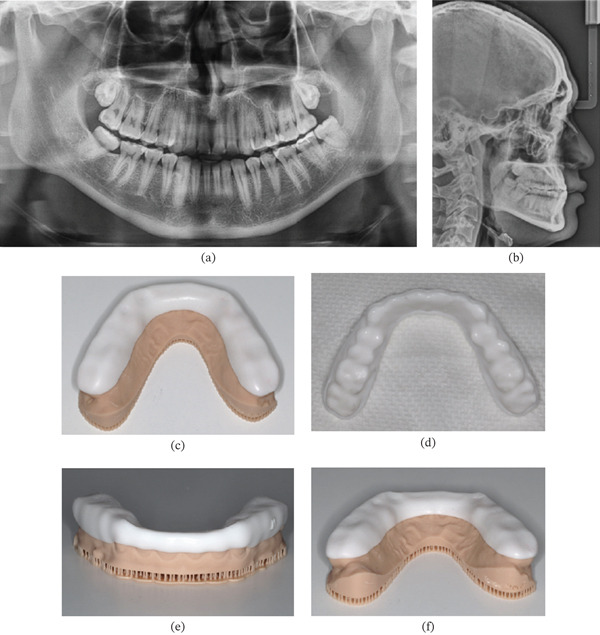
(a) The pre‐treatment panoramic radiograph showed that all permanent teeth were present except 47. (b) The lateral cephalograph demonstrates a mild Class III skeletal relationship base with brachyfacial vertical proportions and slight retroclination of the maxillary incisors. This retroclination was likely the result of the progressive and extensive loss of maxillary anterior tooth structure, both vertically and interproximally. (c–f) A maxillary Michigan‐style nylon occlusal splint (3 mm thickness at the incisal, 1 mm thickness at the posterior occlusal) used to trial new occlusal vertical dimension (OVD).

**Table 1:** Summary of diagnostic findings.

Medical and social history
• Cigarette smoking for approximately 17 years, with a desire to cease this habit
• History of high intake of acidic beverages (e.g., soft drinks, energy drinks and alcohol)
• Significant time spent outdoors in a dusty environment
Skeletal:
• Mild Class III skeletal base relationship with a slightly protrusive mandibular position (Horizontal)
• Brachyfacial vertical morphology (Vertical)
• Normal facial symmetry (Transverse)
Dental:
Posterior relationship:
• RHS Class I molar and canine relationship (Horizontal)
• LHS Class I molar and canine relationship (Horizontal)
• Reduced overall clinical crown height and loss of cuspal anatomy (Vertical)
• Posterior lingual crossbite position of the 14 and 24 (Transverse)
• Group function on RHS and LHS in excursive mandibular movements (Transverse)
Anterior relationship:
• Edge‐to‐edge incisal relationship (Horizontal)
• Contacting on tooth 48 when in protrusion (Horizontal)
• Reduced overall clinical crown heights, reduced anterior overbite and reduced maxillary incisor display on smiling (Vertical)
• Loss of occlusal vertical dimension due to the pre‐existing wear and attrition (Vertical)
• Maxillary dental midline was located 3.0 mm to the right of the facial midline (Transverse)
• Mandibular dental midline was located 1.0 mm to the right of the facial midline (Transverse)
Soft tissues:
• Normal lip competency at rest (Horizontal)
• Normal lower lip position (Horizontal)
• Normal chin point position (Transverse)
Other diagnostic features of note:
• Significant generalised occlusal and incisal edge wear with no reported temporomandibular disorder or current symptoms (patient has a long‐term history of tooth grinding and currently wears an occlusal splint nocturnally).
• Multiple areas of dentine exposure were evident, with an increase in tooth sensitivity reported.
• Dental caries noted in the 17 occlusal surface and in the mesial aspect of the 11
• The panoramic radiograph showed that all permanent teeth excluding the 47 were present and there were large restorations in the 25, 36 and 37 (structurally compromised)
• Asymptomatic and unerupted 18 and 28
• Mild Class III skeletal relationship base with brachyfacial vertical proportions and slight retroclination of the maxillary incisors.
• Mild crowding in the mandibular arch

**Table 2:** Prioritised problem list and ideal treatment objectives.

Prioritised problem list:
• Dietary modification is required, with caries present in teeth 17 and 11
• Significant generalised tooth wear and attrition, which has resulted in an edge‐to‐edge incisal relationship and significant loss of occlusal vertical dimension
• Mild Class III skeletal base relationship (with no significant remaining skeletal growth potential) and retroclination of the maxillary incisors
Ideal treatment objectives:
• Reduce acidic beverages and restore active carious lesions
• Resolve the problematic edge‐to‐edge incisal relationship (i.e., provide positive anterior overjet and overbite) and re‐establish a more ideal and harmonious occlusal vertical dimension through prosthodontic rehabilitation and comprehensive orthodontic treatment. Full mouth rehabilitation to address the functional and aesthetic concerns associated with the pre‐existing tooth wear and attrition.
• Consider removal ± replacement of tooth 37 due to guarded prognosis
• Camouflage the mild Class III malocclusion through a combination of maxillary incisor proclination and clockwise rotation of the mandible through a planned increase in the occlusal vertical dimension
• Contemporary direct adhesive materials to be implemented as medium to long‐term interim restorations as such restorations can be modified as required due to unforeseen issues with patient adaptation or unfavourable stomatognathic parafunction. Eventual conversion from the modifiable composite resin restorations to fixed indirect restorations may be performed once the new OVD is established and is determined to be comfortable for the patient.

**Table 3:** Proposed sequence of interdisciplinary treatment.

1. Preventive treatment, including oral hygiene instruction, dietary modification, smoking cessation therapy, dental prophylaxis and a remineralisation protocol using Tooth Mousse Plus (General Dentist)
2. Occlusal splint to trial an increase in the pre‐existing occlusal vertical dimension (Prosthodontist)
3. Composite rehabilitation at the re‐established occlusal vertical dimension (Prosthodontist)
4. Review the prognosis of tooth 37
5. Comprehensive fixed appliance orthodontic treatment to harmonise the overall occlusion (Orthodontist)
6. Review the post‐orthodontic occlusion and TMJ function. Review the integrity of the composite restorations and the indication for indirect restorations (Prosthodontist)
7. Complete indirect ceramic restorations and new occlusal splint (Prosthodontist)
8. Periodic review of the TMJ function, oral health, restorations, and occlusal splint (General Dentist and Prosthodontist)

## 3. Treatment Progress

An implementation of excellent home care and an improvement in the standard of oral hygiene were prioritised, with emphasis placed on 6 monthly regular dental visits to maintain healthy soft and hard tissues in the oral cavity. A remineralisation protocol (Tooth Mousse Plus, GC) and dietary advice were also provided. Following stabilisation of the oral health condition, a full coverage, Michigan‐style occlusal splint (3 mm thickness at the incisal, 1 mm thickness at the posterior occlusal) was issued by the prosthodontist (Figure 2c–f).

The occlusal splint was worn full‐time for 1 month, excluding eating and cleaning, to test the new OVD. Given that this OVD did not result in any significant issues including TMD, consent was given by the patient to proceed with a composite resin rehabilitation at an increased OVD. Virtual smile design (Digital Smile Design software) was completed using an intra‐oral scan (Trios 4, 3 Shape) and 2D photos (Figure 3). The design was approved by the patient before commencing any further treatment with composite resin.

**Figure 3:**
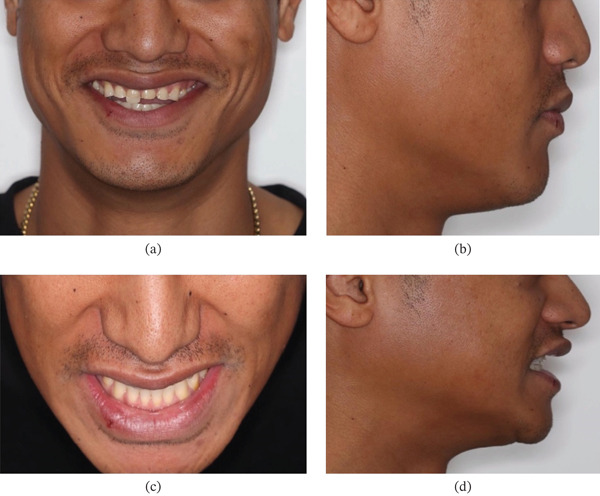
(a) Use of composite resin to increase the vertical height of the maxillary incisor, which provides a guide for the proposed change in occlusal vertical dimension. A smiling photograph was taken to estimate the final incisal edge position. (b) Profile view of the resting lip positions. (c) Smiling photograph taken from a bird’s eye view. (d) The profile view when smiling.

Resin models of the virtual designs were printed and two clear silicone indices with metal separators to maintain the interproximal contacts were made. An injectable composite resin (SE BOND 2, Kuraray Noritake; Gaenial Flow A2, GC) technique was used in conjunction with the clear silicone indices (Exaclear, GC) (Figure 4). Extensive full mouth composite rehabilitation of the maxillary and mandibular teeth was subsequently completed by the prosthodontist (Figure 5). The following bonding protocol was used:

**Figure 4:**
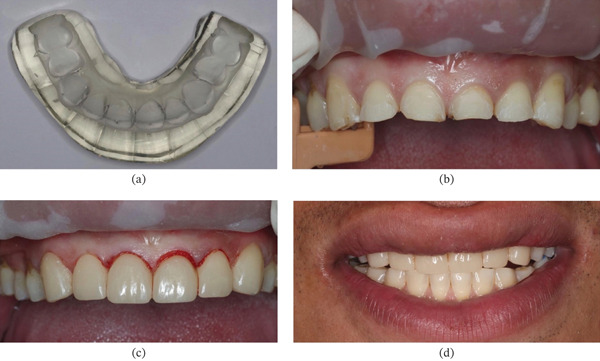
(a) A clear silicone index was used for the injection moulding technique. (b) The maxillary anterior teeth were mechanically prepared. (c) Final adjustment and polishing of the restorations on the maxillary anterior teeth was performed. (d) The new upper incisal length was established and its relationship to lips and mandibular teeth is recorded.

**Figure 5:**
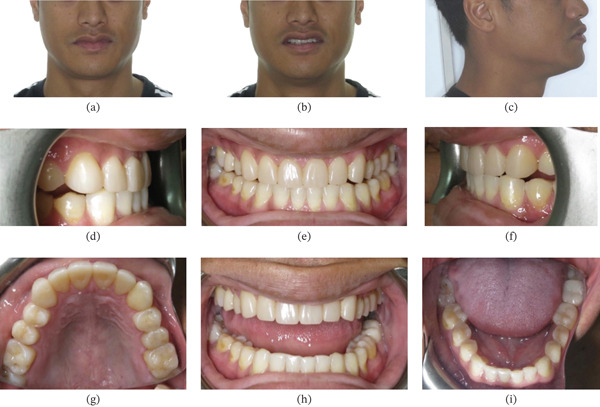
(a–i) Extensive composite resin restorations were provided at the re‐established occlusal vertical dimension. Note the presence of minimal anterior overjet, minimal vertical overbite and bilateral open bites, which were to be addressed with comprehensive orthodontic treatment.

• Air abrasion with 27 *μ*m aluminium oxide particles (Rondoflex, Kavo) was used on the tooth surfaces to be restored

• Selective etching of enamel (Super Etch, Kuraray Noritake)

• SE BOND 2 (Kuraray Noritake)

• Gaenial flowable high strength composite (A2, GC) was injected into the clear silicone indices (Exaclear, GC)

• Final adjustment and polishing was completed with a variety of contouring burs, discs and polishers (Soflex disc, 3 M; Diapolishers; EVE)

The caries was removed from the 17 and the maxillary arch was restored with composite resin according to the previously proposed digital design. The mandibular arch was similarly restored (teeth 35–37, 45, 46). One week later, pre‐adjusted maxillary and mandibular fixed orthodontic appliances (0.022″ × 0.028″ slot, MBT prescription, 3M Oral Care, MN, United States) were bonded (Figure 6).

**Figure 6:**
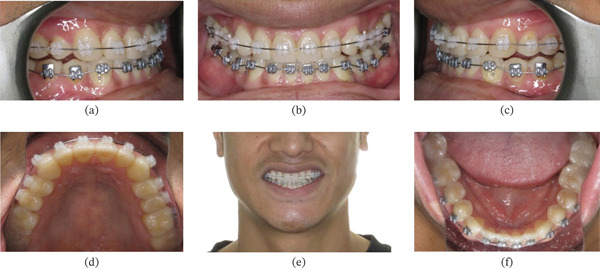
(a–f) Photographs taken immediately following placement of the maxillary and mandibular fixed orthodontic appliances.

After 2 months of active orthodontic treatment, the general dentist made contact with the prosthodontist and orthodontist regarding the compromised 37, which was determined to have a poor long‐term prognosis. Patient was experiencing constant discomfort and sharp sensitivity pain to temperature changes. Cold test came back with a sharp shooting pain with lingering pain more than 30 s. The tooth was diagnosed with symptomatic irreversible pulp. Given that the 38 had clinically erupted, the options of tooth removal or initiating root canal treatment were presented to the patient. Interdisciplinary discussion took place, where the orthodontist explained that complete space closure of the 37 extraction space would be difficult to achieve due to the minimal intra‐arch crowding in the mandibular arch and the brachyfacial vertical morphology. The patient consented to removal of the 37 and was willing to accept a resultant mandibular midline discrepancy and residual extraction space in the posterior third quadrant. It was decided that every effort would be made to provide a harmonious occlusion on the left‐side with maximum antagonistic occlusal interdigitation despite the removal of the 37.

At 13 months, the progress panoramic radiograph (Figure 7a) demonstrated incomplete closure of the 37 extraction space and pleasing overall root parallelism and minimal external root resorption was noticed. The progress lateral cephalogram demonstrated therapeutic proclination of the maxillary incisors and slight clockwise rotation of the mandibular plane angle (Figure 7b). Progress photographs were taken 14 months into active treatment (Figure 8), which demonstrated the improved maxillary and mandibular arch alignment. As expected, the maxillary to mandibular dental midline discrepancy (i.e., located to the patient’s left‐side) had increased in dimension following the removal of the 37.

**Figure 7:**
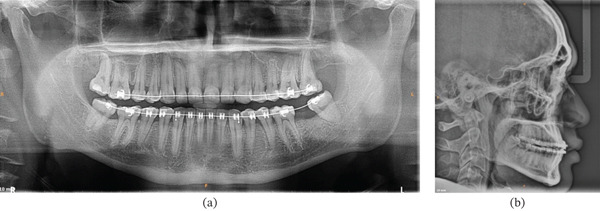
(a) The progress panoramic radiograph taken 13 months into active orthodontic treatment demonstrated incomplete closure of the 37 extraction space and pleasing overall root parallelism. (b) The progress lateral cephalograph demonstrated therapeutic proclination of the maxillary incisors and slight clockwise rotation of the mandibular plane angle.

**Figure 8:**
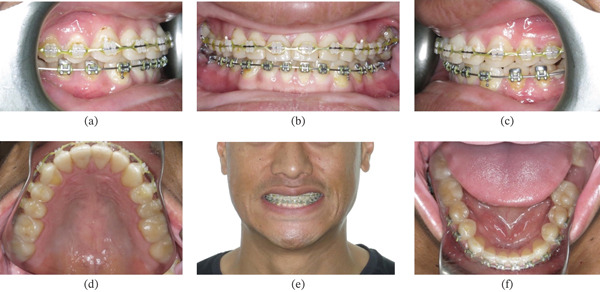
(a–f) Photographs taken 14 months into active orthodontic treatment demonstrated incomplete closure of the 37 extraction space and an increase in dimension of the maxillary to mandibular dental midline discrepancy.

After 20 months, the fixed labial orthodontic appliances were removed (Figure 9). Maxillary and mandibular vacuum‐formed Essix C+ material (Dentsply Raintree Essix) removable retainers were also issued for interim nocturnal wear. The orthodontist noted that the palatal aspect of the direct adhesive restoration on the 13 had recently fractured and subsequently advised the prosthodontist.

**Figure 9:**
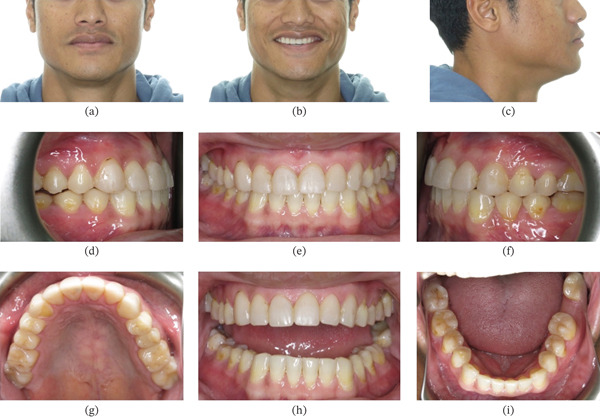
(a–c) Extra‐oral photographs taken at the completion of 20 months of active orthodontic treatment. The anterior smile and facial profile aesthetics are pleasing. (d–i) The overall anterior and posterior occlusal relationships appear harmonious following this carefully sequenced interdisciplinary treatment.

The orthodontist instructed the patient to wear the removable retainers nocturnally, which would provide protection of the direct adhesive restorations until the prosthodontist could re‐evaluate the occlusion and plan the final indirect restorations.

The patient was reviewed by the prosthodontist and it was recommended to place more durable indirect restorations on the more significantly structurally compromised teeth (i.e., 13 to the 23, 25, 36, 45 and 46). The patient was informed of the procedures required, along with the potential benefits and possible complications that may occur.

After the provision of local anaesthesia, the maxillary anterior teeth, along with teeth 25, 35, 36, 45 and 46 were prepared for conservative indirect restorations (Figure 10). As the patient did not have extremely high aesthetic expectations, monolithic restorations with minimal buccal layering porcelain were recommended, as such restorations would only require conservative tooth preparation and should provide excellent durability [3]. The patient also agreed to accept supragingival crown margins where possible, despite the likelihood of their visibility. Lithium disilicate was used for the posterior onlay restorations, as the bond strength to enamel over time is generally both strong and reliable [4]. High accuracy polyvinyl siloxane impression material (Light and heavy body, Flexitime, Heraeus Kulzer) was used.

**Figure 10:**
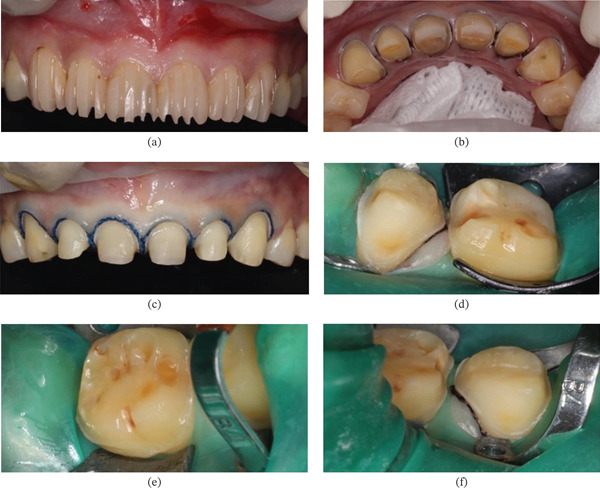
(a) Labial view of the reduction grooves, which were cut to guide the conservative crown preparation for the maxillary anterior teeth. (b) Occlusal view of the completed conservative crown preparation for the maxillary anterior teeth. (c) Retraction cords were placed in the gingival sulcus prior to taking an impression. (d–f) Rubber dam was placed prior to the cementation of the crowns for the mandibular posterior teeth.

Zirconia crowns (IPS e.max ZirCAD Prime, Ivoclar) were fabricated, fitted, adjusted and cemented to the maxillary teeth (13 to the 23, 25) with self‐etching primer/bond and self‐adhesive dual‐cure resin cement (UBQ, Panavia SA Universal, A2), as per the manufacturer’s instructions. Monolithic lithium disilicate crowns (IPS. E.max, Ivoclar) were cemented to the mandibular teeth (35, 36 and 45, 46) with self‐etching primer/bond and self‐adhesive dual‐cure resin cement (UBQ, Panavia SA Universal, A2) (Figure 11). After insertion of crowns, lateral excursion with canine guidance and protrusion with anterior contact and simultaneous posterior disocclusion were evaluated. Phonetics of ‘T’, ‘S’ and ‘V’ sounds and lisps were assessed, and adjustments of occlusion were made as necessary. An occlusal splint (3D printed nylon, 3D Sleep) was made for the patient to wear nightly and a mouthguard (EVA) during engagement in contact sports. The temporomandibular joint function and occlusion, the integrity of the dentition and the surrounding supporting tissues were reassessed 1‐month post‐insertion and determined to be sound. The facial and smile aesthetics were pleasing and the patient expressed satisfaction with the overall process and the outcome achieved.

**Figure 11:**
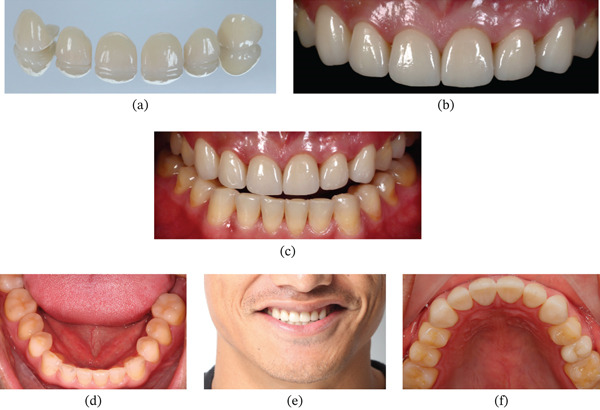
(a) The maxillary anterior zirconia crowns prior to cementation. (b–c) The maxillary anterior zirconia crowns following their cementation. (d) Maxillary occlusal photograph demonstrating the zirconia crowns on the maxillary anterior teeth and the 25. (e) The static smiling photo showcases the pleasing anterior hard and soft tissue aesthetics. (f) The lithium disilicate crowns were placed on teeth 35, 36, 45 and 46.

Regular dental recall appointments were done every 6 months and specialists follow‐up were done yearly. An evaluation of TMJ function, restoration margin integrity and periodontium health together with reinforcement of oral hygiene instruction and diet analysis were done during each recall appointment. Pulp status of the indirect restoration teeth was assessed through history‐taking, percussion test. Follow‐up radiographs will be taken every 2 years. During the recall appointment, patient acknowledged the treatment had given him a new smile with the ability to eat and drink comfortably without fear of experiencing sensitivity.

## 4. Discussion

Tooth wear is becoming more prevalent in adolescents and young adults, and shows significant correlation with dietary habits and lifestyle [5]. A PubMed search for ‘tooth erosion’ (MeSH) revealed over 100 studies published in 2000—twice as many as in 1980 [6]. In clinical practice, the extended longevity of teeth place greater demands on the preventive and restorative abilities of dental professionals to manage the clinical effects of severe tooth wear [7].

The aetiology of tooth wear is multifactorial, with acid erosion, attrition and abrasion all potential factors that can contribute to tooth wear [8]. Figure 12 outlines endogenous and exogenous contributors. This case showed occlusal erosion and non‐carious cervical lesions, leading to sensitivity, pulpal risk and aesthetic issues [5].

**Figure 12:**
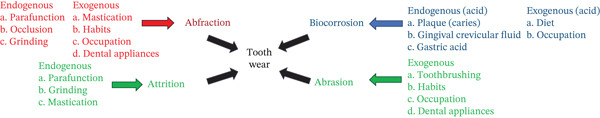
Potential contributing factors to tooth wear.

## 5. Diagnosing Tooth Wear

Severe tooth wear is defined as tooth wear resulting in a substantial loss of tooth structure, with dentine exposure and significant loss (i.e., one‐third or more) of the clinical crown [9]. Tooth wear is a normal physiological process, unless it causes pain, sensitivity, functional loss or aesthetic concerns—then it becomes pathological. Not all severe wear is pathological; for example, age‐related wear in elderly patients may be considered normal. Therefore, a comprehensive examination and history‐taking are crucial for appropriately diagnosing and managing tooth wear. Index scores such as the Tooth Wear Index (TWI), Basic Erosive Wear Examination (BEWE) or the Tooth Wear Evaluation System (TWES 2.0) are tools that can be applied by clinicians to determine the severity of tooth wear and quantify its progression [10] [11] [12]. It is important for clinicians to appreciate the advantages and disadvantages of the various available assessment tools and to determine the most appropriate diagnostic screening tool for an individual patient.

## 6. OVD and Tooth Wear

In general, tooth wear progresses relatively slow, with a typical loss estimated to be less than 15 microns per year [13]. When tooth wear progresses at a slow rate, compensatory eruption of maxillary and mandibular teeth occurs [14]. Because of this compensatory effect, only minimal changes in the actual OVD occur. For that reason, careful consideration should be taken when changing an individual’s OVD to create restorative space. A review by Abduo and Lyons stated that increasing OVD by up to 5 mm intrinsically can be safe and predictable when performed appropriately [15]. Although tooth wear is often linked to temporomandibular disorders (TMD), studies show no direct association between sleep bruxism and TMD [16]. Attrition also does not increase TMD prevalence [17] [18].

## 7. Prosthodontic Management

Managing generalised wear requires a simple, adjustable and cost‐effective approach. A recommended protocol includes the following:

• Establishing TMJ and occlusal position
• Planning OVD increase
• Diagnostic wax‐up
• Interim restorations
• Final restorations
• Follow‐up [19]

Centric occlusion (CO) and centric relation (CR) are preferred for their reproducibility [20]. For significant changes in OVD, an occlusal splint can be provided to trial the new OVD and TMJ responses before any irreversible treatment is provided. A nylon splint was chosen for this case due to its biocompatibility, flexibility and high precision making it comfortable to wear while maintaining a good mechanical strength [21].

## 8. Orthodontic Management

The objectives of orthodontic treatment for management of patients with tooth wear may include the alteration of occlusion to maintain or increase the OVD, to improve the inter‐incisal relationship, reposition abutments, to close or open edentulous spaces and to enhance soft tissue periodontal aesthetics. Visualisation of the final therapeutic occlusal relationship helps assess the specific space and anchorage requirements for the proposed orthodontic tooth movements [22]. The orthodontist and prosthodontist will also need to determine whether orthodontic treatment is required to create anteroposterior and/or vertical space for the proposed restorative treatment [23]. Uneven gingival margins are common during compensatory passive eruption of teeth, which can negatively affect the anterior smile aesthetics. Selective orthodontic detailing can provide symmetrical and harmonious gingival aesthetic contours for the anterior teeth [14] [24]. The final therapeutic position of the maxillary and mandibular incisors is crucial for achieving pleasing smile aesthetics and to enhance post‐treatment stability [25–27].

## 9. Material of Choice

Modern composite resins, when bonded properly, offer reliable full‐mouth rehabilitation with a 2.3% failure rate over 5.5 years [28]. In this case report, the palatal composite on the maxillary right canine fractured approximately after 21 months likely due to insufficient thickness and low flexure strength of flowable composite [29]. Flowable composite resin with nanoparticle (nanosilica) filler increases the flexure strength and provides better fracture resistance reducing the chance of fracture that could be an alternative material in future cases [30]. Additionally, ceramic restorations provide higher compression and flexural strength compared with composite resin. It is proven to be more effective in restoring severely worn and damaged teeth [31]. Because of previous history of erosion and abrasion, zirconia and ceramic restorations were chosen as the final restorations. After the completion of the final restorations, an occlusal splint is often recommended for patients who have history of parafunctional habits, to protect the restorations and teeth from wear and fracture [32].

## 10. Cementation Materials

The long‐term bonding durability of fixed prosthodontic restorations requires an understanding of compatibility between the cementing agent and the restoration material. It has been reported that airborne particle abrasion (110 *μ*m Al_2_O_3_ at 2.5 bar) followed by cementation with a phosphate monomer dual‐cured resin cement provides a durable resin bond to zirconium oxide ceramic [33]. Other studies have showed that the highest bonding strength for leucite‐reinforce feldspathic porcelain and lithium‐disilicate glass ceramic restorations result from a surface treatment with hydrofluoric acid (HF) followed by a saline coupling agent [34–36]. Conventional multi‐step resin bonding (i.e., etch and rinse, self‐etch adhesive or priming) results in higher adhesive bond strengths in comparison with self‐adhesive resin cement [36–38]. To improve the bond strength, a self‐adhesive, dual‐polymerising universal resin cement (SARC) (Pavania SA Universal, Kuraray Noritake) was used in conjunction with a self‐etching primer (UBQ, Kuraray Noritake) in this case report. This combination, along with surface pre‐treatment, offers high bond and shear strength [36] [39].

## 11. Reflection

Following this case report, diagnosing the aetiology of pathological occlusal wear is crucial in the prevention of tooth wear progression and for the stability of final restoration after rehabilitation treatment. The importance of disease control and prevention by educating patient with the risk factors of occlusal wear is an important step before restorative treatment. Stabilisation of occlusal scheme is required before definitive restoration. Selection of provisional and final restoration material should be based on occlusion scheme and aetiology of tooth wear. Multi‐disciplinary collaboration is recommended to achieve optimal and minimal invasive outcome in a complex case.

## 12. Conclusion

This case report demonstrates the importance of comprehensive diagnosis and appropriate interdisciplinary management for an adult patient with severe tooth wear. Tooth wear is a common issue that may negatively affect both function and aesthetics. Comprehensive diagnosis is essential to develop the relevant treatment options, and to identify the respective risks, costs, benefits, and limitations of each treatment option. Discussing realistic treatment objectives and the outlining the predictability of the available treatment options are also important components of the informed consent process.

**Abbreviations**
GERD: gastroesophageal reflux disease
OVD: occlusal vertical dimension

## Author Contributions

P. J. Un: conceptualization; investigation; project administration; validation; visualization; writing – original draft; writing – review and editing.

D. C‐V. Ong: investigation (provided clinical data and completed treatment); supervision; validation; writing – original draft; writing – review and editing.

Jamie Foong: investigation (provided clinical data and completed treatment); validation; writing – review and editing.

## Funding

No funding was received for this manuscript. Open access publishing facilitated by The University of Queensland, as part of the Wiley ‐ The University of Queensland agreement via the Council of Australasian University Librarians.

## Disclosure

All authors have read and approved the final version of the manuscript. Dr. Desmond Ong and Dr. Jamie Foong had full access to all of the data in this study and takes complete responsibility for the integrity of the data and the accuracy of the data analysis.

## Ethics Statement

A written informed consent was obtained from the patient for the publication of this case report. As a single‐case report with the patient’s signed consent, no other ethical review was required.

## Conflicts of Interest

The authors declare no conflicts of interest.

## Data Availability

Data sharing not applicable to this article as no datasets were generated or analysed during the current study.
